# Characterisation of heritable *TP53*-related cancer syndrome in Sweden—a nationwide study of genotype-phenotype correlations in 90 families

**DOI:** 10.1038/s41431-024-01753-1

**Published:** 2025-01-05

**Authors:** Meis Omran, Yaxuan Liu, Alexander Sun Zhang, Anna Poluha, Marie Stenmark-Askmalm, Fredrik Persson, Anna-Lotta Hallbeck, Anna Rosén, Hafdis T. Helgadottir, Emma Tham, Svetlana Bajalica-Lagercrantz

**Affiliations:** 1 Department of Oncology-Pathology, Karolinska Institutet, BioClinicum, SE-171 77 Stockholm, Sweden; 2 Cancer Theme, Karolinska University Hospital, SE-171 76 Stockholm, Sweden; 3 Department of Breast Surgery, Obstetrics and Gynecology Hospital of Fudan University, Shanghai, 200011 China; 4 Department of Clinical Genetics, Genetics and Pathology, Uppsala University Hospital, SE-751 85 Uppsala, Sweden; 5 Department of Immunology, Genetics and Pathology, Uppsala University, SE-751 05 Uppsala, Sweden; 6 Division of Clinical Genetics, Department of Laboratory Medicine, Office for Medical Services, Skåne University Hospital, SE-228 85 Lund, Sweden; 7 Department of Clinical Genetics and Genomics, Sahlgrenska University Hospital, SE-413 45 Gothenburg, Sweden; 8 Department of Clinical Genetics, Linköping University Hospital, SE-581 85 Linköping, Sweden; 9 Department of Diagnostics and Intervention, Oncology, Umeå University, SE-901 87 Umeå, Sweden; 10 Department of Molecular Medicine and Surgery, Karolinska Institutet, Stockholm, SE-171 76 Sweden; 11 Clinical Genetics and Genomics, Karolinska University Hospital, SE-171 76 Stockholm, Sweden

**Keywords:** Medical research, Health care

## Abstract

We aimed to describe the clinical characteristics of families with heritable *TP53*-related cancer (h*TP53*rc) syndrome in Sweden with class 4 and 5 germline *TP53* variants (g*TP53*), and to evaluate the genotype-phenotype correlation. These results were also used to evaluate our previously published phenotype prediction model based on *TP53* missense variants and their impact on protein conformation. 90 families with h*TP53*rc were initially identified in Sweden. After variant reclassification using the *TP53*-specific ACMG criteria, 83 families remained (176 carriers) to harbour a pathogenic (class 5) or likely pathogenic (class 4) variant in *TP53*. Of these, 112 carriers (64%) had a previous history of cancer, and 35 (31%) had developed more than one primary tumour. 16% of the families met the stricter criteria for Classic Li-Fraumeni syndrome, 45% the updated Chompret criteria, 35% for hereditary breast cancer (HBC), and the remaining 5% were classified as “Others”. We identified 42 different g*TP53* variants of which 22 were missense. The most frequently observed variant was the missense c.542 G > A, p.R181H identified in 14/29 (48%) of HBC families. Fifteen of the 20 informative missense variants (75%) were phenotypically predicted correctly using our previously published in silico prediction model. The *TP53* p.R181H was identified as a common Swedish variant predominantly associated with an HBC phenotype. Apart from this variant, there were no significant genotype-phenotype correlations. Therefore, due to phenotypic overlap it is still too early to stratify surveillance programme for different *TP53*-carriers.

## Introduction

Heritable *TP53*-related cancer (h*TP53*rc) syndrome, also known as the Li-Fraumeni syndrome (LFS), is characterized by a high risk of developing mainly four core tumours; early onset breast cancer (before 31 years of age), sarcomas, malignant brain tumours and adrenocortical carcinomas [1]. The early reports of LFS constituted mainly of families representing a severe phenotype with early onset of tumours, but with increased germline testing on a wider indication, a more complex picture has emerged. Currently, there is an ongoing discussion about the phenotypic spectrum among families with germline *TP53* (g*TP53*) variants and the challenges in genetic counselling and clinical handling. Clinically, some families appear to be more prone towards developing childhood tumours, while other express a higher risk for different cancer types mainly as adults. In addition, some families tend to be mostly predisposed to develop breast cancer only. *TP53*-carriers develop cancer at substantially younger ages than non-carriers with the same cancer types [2, 3]. A recent paper estimated that *TP53*-carriers have a nearly 24 times higher incidence of any cancer than the general population, especially from childhood to 30 years of age [4]. Women with a likely pathogenic or pathogenic (class 4 or 5) *TP53* variant appear to have a generally higher cancer risk than male carriers, not only due to the increased risk for breast cancer, but they also tend to have an overall higher cancer incidence [2, 3, 5, 6]. In 2020, The European surveillance recommendations were published, suggesting surveillance including whole-body MRI for all individuals with h*TP53*rc for earlier cancer detection [7].

Despite reports on genotype-phenotype correlations, such as the Brazilian founder variant p.R337H and the predominance of adrenocortical carcinomas, surveillance recommendations are still not personalized based on genotype, thus all carriers are recommended the same surveillance. However, larger cohort studies on *TP53*-carriers may provide data for surveillance stratifications according to family risk profiles in the future.

Here, we provide a nationwide genetic and clinical characterisation of carriers of pathological and likely pathological g*TP53* variants based on data from their pedigrees at diagnosis and genetic testing reports.

## Material and methods

### Data collection and sources

Data were retrospectively collected through the six hereditary cancer units in Sweden i.e. at the University Hospital in Umeå, Akademiska University Hospital (Uppsala), Karolinska University Hospital (Stockholm), Linköping University Hospital, Sahlgrenska University Hospital (Gothenburg) and Skåne University Hospital (Lund). All individuals, regardless of age, with a pathogenic (class 5) or likely pathogenic (class 4) *TP53* variant were included since the testing started at each participating centre with the earliest accessible data from January 2000 and up until March 2022. All patients fulfilling either Classic LFS or Chompret criteria during this period were offered testing for germline *TP53*. During April 2012-April 2018 all cancer genetic units in Sweden offered genetic screening for families with suspected hereditary breast and ovarian cancer, including *TP53*, within a national study SWEA (The Swedish *BRCA1/2* Extended Analysis) [8] later resulting in a broader clinical screening panel for HBC including *TP53*. Therefore, the latter period of testing (2012-2022), had a wider indication for *TP53* screening. Screening criteria for hereditary breast and ovarian cancer used within SWEA is outlined in Table S1. Since 2018, *TP53* is included in the hereditary breast cancer panel in Sweden.

Pedigrees that were used within the clinical investigation for genetic counselling for hereditary *TP53* variants at each site were obtained for all individuals and served as a base for registering cancer diagnoses and family phenotypes including gender, tumour type, age of tumour onset, number of primary tumours and, when available, histopathological analysis report of tumours. Cancer diagnoses were in most cases confirmed for index patients and first-degree relatives at the time of genetic counselling. Data on variant testing included sample tissue, date of testing, variant type and original variant classification. Variants confirmed to be clonal haematopoiesis of indeterminate potential (CHIP) through normal analysis of a second tissue were excluded. All data were compiled in a registry within REDCap (version 11.1.15) at the coordinating site at Karolinska Institutet, Stockholm. Only individuals confirmed to be carriers of a pathogenic or likely pathogenic germline *TP53* variant are in the registry, thus deceased relatives with cancer, but without confirmed carriership, are not included. All families were classified as either fulfilling the Classic Li-Fraumeni syndrome (Classic LFS) criteria [1], the revised Chompret criteria [9], HBC criteria or, when genetic screening was performed outside any of the mentioned criteria, as “Other” in May 2023 (see Table S2).

### Variant classification and prediction

All variants were classified according to the original American College of Medical Genetics and Genomics/the Association for Molecular Pathology (ACMG/AMP) criteria [10] from 2015, and by the *TP53*-specific classification criteria [11] published in 2021. The classification was made by four researchers (MO, YL, ASZ, ET,). Heterozygous variants were considered to be de novo if both parents were tested and neither were carrier (in clinical routine, no ID-confirmation of parenthood is performed, and thus this data was not available). All mosaic variants were considered to be de novo somatic mosaicism if clonal haematopoiesis was excluded through testing of a second tissue and/or if the individual had a child who inherited the variant. If no second tissue was available (and no child inherited the variant), the mosaic variants were considered to be probably de novo. Data from the tp53 mutation database (https://tp53.isb-cgc.org) was collected as part of this classification including the DNE_LOF column as defined by Fortuno et al. [11]. In the cumulative cancer incidence analysis, all truncating variants were classified as notDNE_LOF and variants without classification were not included in the analysis. All missense variants were also evaluated by the phenotypic prediction model previously published by the group [12], a logistic regression model that was developed to predict LFS and HBC outcomes, based on the effect of individual *TP53* missense variants and their impact on TP53 protein conformation.

### Statistical analysis

Data were collected and processed using the R software (version 4.2.2), in RStudio (version 2022.12.0 Build 353). The log-rank test was used to calculate differences in cumulative cancer incidence. Continuous variables from two entities (two different groups) were evaluated by unpaired, two-tailed t test or Mann-Whitney test, and continuous variables from three entities (i.e. three types of families) were compared by using ANOVA analysis or Kruskal-Wallis test. Statistical significance was set at *p*$\le$0.05.

## Results

### Germline *TP53* variants and genotype-phenotype correlation

In total, 90 families with initially clinically actionable g*TP53* variants were originally identified.

After reclassification of the variants using the 2021 germline *TP53* classification criteria [11]. In addition, one case was confirmed to be a CHIP after negative fibroblast sequencing and was thus excluded from the cohort. Three variants (one missense and two in-frame deletions) in three families were reclassified to variants of unknown significance (VUS) and one missense variant found in four families was reclassified to likely benign. Seventy-two percent (33/46) of the *TP53* variants remained unchanged after reclassification using the original ACMG/AMP criteria from 2015 [10] compared to the g*TP53*-specific ACMG/AMP criteria from 2021 [11]. Only in-frame variants (in-frame deletions and missense variants) were downgraded to benign or uncertain significance (see Table 1). The summary of criteria used for the g*TP53* specific ACMG/AMP classification of all variants is listed in the Table S5.

**Table 1:** Clinical characteristics and variants of families in Sweden with heritable *TP53*-related cancer syndrome.

*UTR* untranslated region, *TAD* transactivation domain, *PRD* proline-rich domain, *DBD* DNA binding domain, *LR* linker region, *OD* Oligomerisation domain.

^#^The possibility of a germline *TP53* variant carrier developing LFS instead of HBC with a threshold value 0.65; *Predicted pheotype in agreement with presented phenotype. **TP53-specific criteria (Fortuno et al.)^11^.

^$^Phenotype presented as Classic LFS/Chompret if the number of Classic LFS/ Chompret families is larger than the number of HBC families, and if vice versa, the phenotype presented as HBC.

^§^Non informative was used when the number of Classic LFS/Chompret families is equal to the number of HBC families or Other.

*DNE* dominant-negative variant, *LOF* loss of function,  indicate variants that have been reclassified to 2 or 3 and thus, removed from the analysis; **Bold** indicate variants upgraded after reclassification; *Italic* indicate variants down-graded after reclassification.

**Fig. 1:**
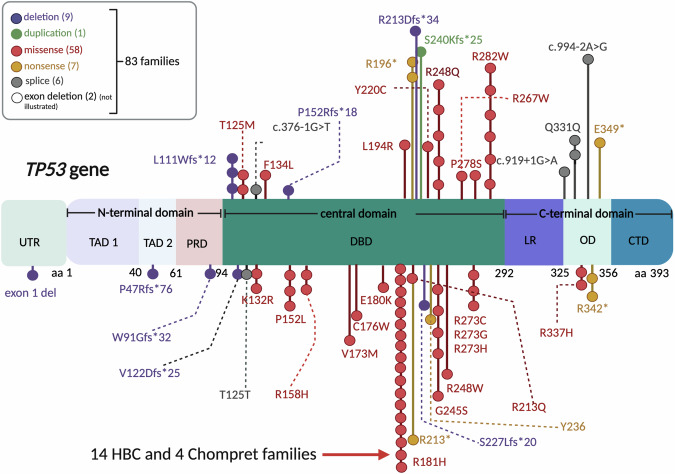
Linear structure of the TP53 protein, with the 42 different variants in the Swedish cohort. Colour coding indicates type of variants identified in families; numbers specified in parentheses. Each dot corresponds to one family. The deletion in family 226 (exon 1) and the large deletion in family 15 (exon 2–9) are not illustrated in the figure. CTD = carboxyl terminus domain, regulatory domain enhancing the binding to DNA. DBD DNA binding domain, LR linker region, PRD proline rich domain, promoting p53-programmed cell death and growth suppression, OD oligomerization domain, TAD transactivation domain, TAD 1 is linked to transcriptional activity, and TAD 2 to MDM2 binding. Amino acid abbreviations: C cysteine, G glycine, H histidine, L leucine, M methionine, P proline, R arginine, S serine, T threonine, W tryptophan, Y tyrosine. * = stop codon.

Thus, 83 families with in total 42 different pathogenic or likely pathogenic variants remained classified as h*TP53*rc. These 42 variants consisted of 22 missense, 8 frameshift deletions/duplications, 2 large deletions, 5 nonsense, and 5 splice variants. Of the 8 frameshift variants, only one (p.R213Dfs*34) had been previously reported, in France [13]. Most of the variants were located in the DNA-binding domain (Table 1, Fig. 1). Among these, the most commonly occurring variants were p.R181H identified in 18 families (Chompret 4, HBC 14), p.R282W carried by six families (LFS 1, Chompret 4, HBC 1), p.G245S and p.R248Q harboured by five families (Classic LFS 1, Chompret 2, Other 2; Classic LFS 1, Chompret 3, HBC 1) respectively (Table 1).

### Clinical category of families with h*TP53*rc and age of first tumour onset

**Fig. 2:**
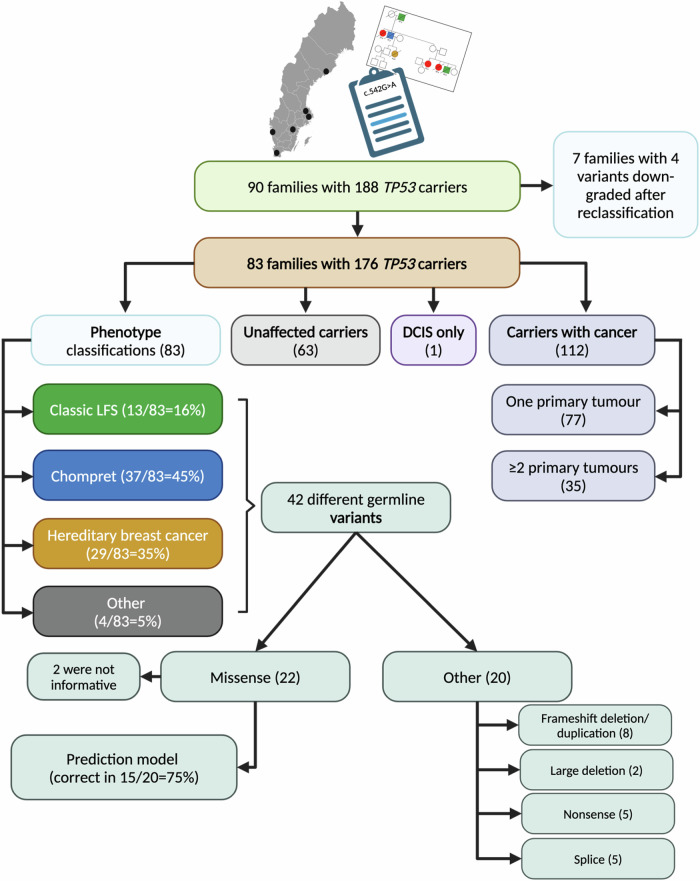
Flow-chart of the different phenotypes, carriers and variants. The prediction model was only used on missense variants to predict the phenotype to be either LFS/Chompret or hereditary breast cancer.

**Fig. 3:**
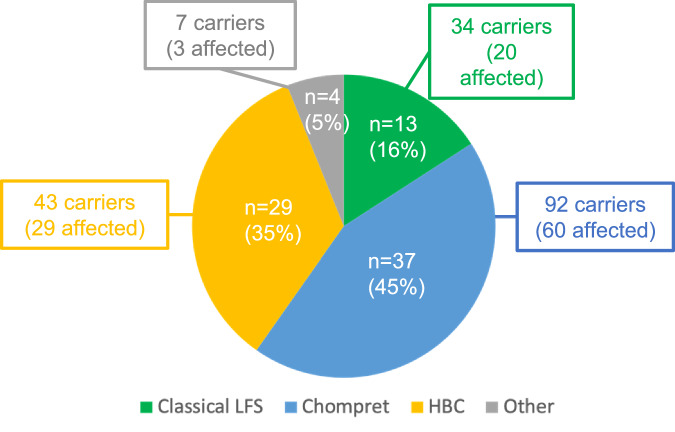
The clinical category of 83 families with h*TP53*rc in Sweden and the age of first tumour onset in affected germline *TP53* variant carriers. The number (n) and percentage of Classic LFS, Chompret, HBC and “Other” families. Germline *TP53* variant carriers are identified from these four types of families, respectively, and affected carriers are carriers with at least one malignant tumour or premalignant lesion.

Of the 83 pedigrees, 13 (16%) met the more stringent Classic LFS criteria for genetic screening, 37 (45%) fulfilled the wider Chompret criteria, and 29 (35%) met the HBC criteria. In addition, four families (5%) were identified after *TP53* screening outside the mentioned criteria and were thus assigned to the group “Other” (Figs. 2, 3; Table 2). These families were characterised by a single proband in each family with the following phenotypes: hypodiploid acute lymphoblastic leukaemia at the age of 13 (patient 80); bilateral ductal breast carcinoma in situ (DCIS) at the age of 26 (patient 72_203); soft tissue sarcoma and oesophageal adenocarcinoma, both at the age of 47 (patient 66); and ovarian cancer at the age of 62 (patient 108; Table S3). Notably, patient 108 was also a carrier of a pathogenic germline *BRCA1* variant as well as the g*TP53* variant.

**Table 2:** Tumour types and average age of tumour onset in the 112 patients out of 176 germline *TP53* variant carriers.

			Classic LFS		Chompret		HBC		Other	
Tumour types	No. of malignancies	No. of patients	No. (%)	mean/median age	No. (%)	mean/median age	No. (%)	mean/median age	No. (%)	mean/median age
				at tumour onset (range)		at tumour onset (range)		at tumour onset (range)		at tumour onset (range)
LFS core cancer									0	–
Breast cancer	81	71	7 (6.2)	35/32 (25-47)	38 (33.6)	35/33 (23–71)	26 (23.0)	43/37 (30–69)	0	–
Soft tissue sarcoma	14	13	7 (6.2)	26/25 (2–49)	4 (3.5)	51/47 (38–65)	1 (0.09)	63 (60–65)	1 (0.09)	47
Osteosarcoma	6	6	2 (0.017)	13 (8–17)	4 (3.5)	29/30 (12–46)	0	–	0	–
CNS tumour	16	16	4 (3.5)	17/13 (7–35)	11 (9.7)	29/28 (0.3–53)	1 (0.09)	71	0	–
Adrenocortical carcinoma	5	5	0	–	5 (4.4)	3/2 (1–5)	0	–	0	–
Other cancer									0	–
Stomach carcinoma	7	7	2 (0.017)	49 (46–52)	3 (2.7)	57/64 (42–68)	2 (0.17)	40 (40–40)	0	–
Lung cancer	5	5	2 (0.017)	44 (43–45)	3 (4.7)	45/42 (41–52)	0	–	0	–
Ovarian carcinoma	5	5	1 (0.09)	25	1 (0.09)	47	2 (0.17)	68 (60–75)	1 (0.09)	62
Melanoma	4	4	2 (0.017)	27 (19-35)	1 (0.09)	56	1 (0.09)	54	0	–
Prostate cancer	3	3	1 (0.09)	50	1 (0.09)	38	1 (0.09)	45	0	–
Colorectal cancer	4	4	0	–	4 (3.5)	38/41 (31–52)	0	–	0	–
Leukaemia	3	2	0	–	1 (0.09)	11	0	–	1 (0.09)	14/14 (13;15)**
Thyroid carcinoma	3	3	1 (0.09)	34	2 (0.017)	24 (22–26)	0	–	0	–
Cervical cancer	1	1	0	–	1 (0.09)	22	0	–	0	–
Skin cancer	1	1	0	–	1 (0.09)	61	0	–	0	–
Pancreas carcinoma	1	1	0	–	0	–	1 (0.09)	63	0	–
Oesophageal cancer	1	1	0	–	0	–	0	–	1 (0.09)	47
Liver	1	1	0	–	0	–	1 (0.09)	40	0	–
Renal cancer	1	1	0	–	0	–	1 (0.09)	54	0	–
Unkown primary cancer with multiple metastases	2	2	1 (0.09)	42	1 (0.09)	67	0	–	0	–
Total amount of cancers	**164**									
Premalignant lesions										
Basalioma^a^	6	5	1 (0.09)	46	4 (3.5)	47/48 (31–59)	1 (0.09)	57	0	–
DCIS	5	5	1 (0.09)	22	1 (0.09)	27	3 (8.6)	56/63 (37-67)	0	–
Cervical cancer in situ	1	1	0	-	1 (0.09)	36	0	–	0	–

^a^Basalioma is treated as a premalignat lesion; “-“ not applicable; No% indicate proportion of all 112 affected carriers.

^b^The same patient developed different leukaemia diagnoses twice, at 13 and 15 years, respectively.

Of the 176 *TP53* carriers with a class 4/5 variant, 157 were adults ≥18 years (111 women and 46 men) and 19 were children (10 girls and 9 boys) at the date of genetic testing. 112 (64%, 84 females and 28 males) had developed at least one malignant tumour (20 from Classic LFS families, 60 from Chompret families, 29 from HBC families and 3 from “Other” families) and a total of 164 tumours were diagnosed (Fig. 3, Table S3).

### Unaffected carriers

Sixty-three of the carriers (37 women and 26 men) were unaffected at contact with the cancer genetic unit and the pedigree establishment. The mean age at genetic analysis was 40 years with a range from 2 to 71 years. Fourteen unaffected carriers were from Classic LFS families with the mean age of 22 years (range 6–41 years). Thirty carriers were unaffected among the Chompret families with the mean age of 37 years (range 4–64 years), and sixteen unaffected carriers from HBC families with a mean age of 47 years (range 2–71 years).

### Tumour types and frequency of multiple primary tumours

The different tumour types identified in the four family groups are specified in Table 2 and more extensively in Table S3. The most commonly occurring tumour type was breast cancer (Table 2). The mean age of breast cancer onset in HBC families (43 years) was significantly older compared to Chompret families (35 years, *p* = 0.02) and to Classic LFS (34.6 years), but no statistical difference was found between Classic LFS and Chompret (*p* = 0.18). Two childhood soft tissue sarcomas were found, all among Classic LFS families (Table S4), four patients with colorectal cancer (Table 2), all within Chompret families, and five patients with only DCIS were found, three within HBC families, one in LFS and one in Chompret families, respectively (Table 2). Two patients with leukaemia of which one was found in a Chompret and one in an “Other” family. No childhood tumours were found among HBC families (Table S4).

**Fig. 4:**
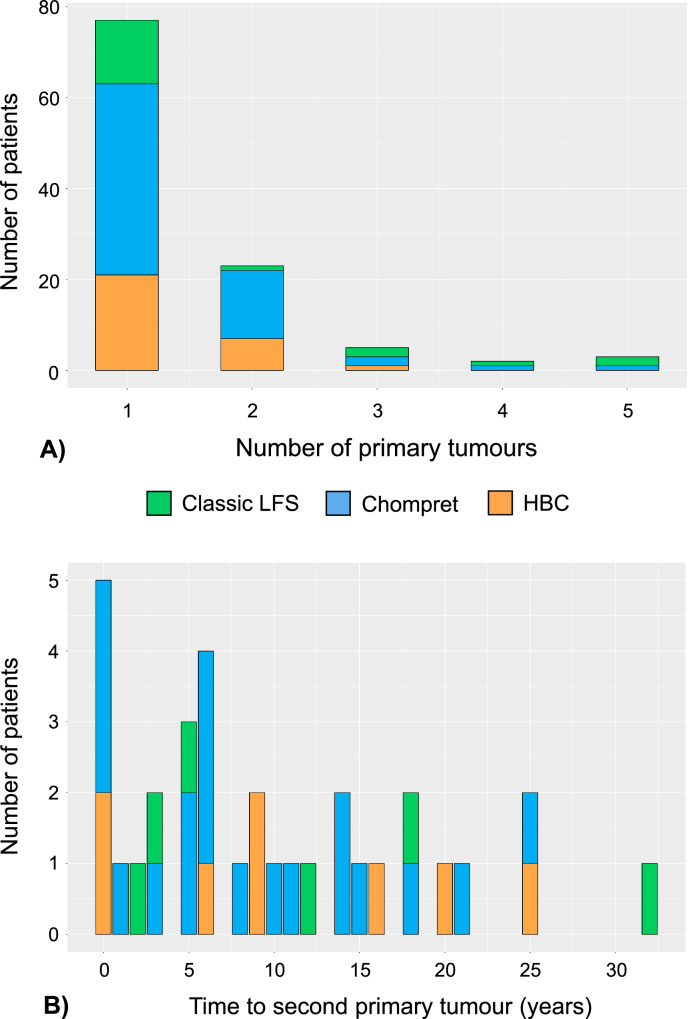
The number of primary tumours and time to second primary tumour in 33 patients with multiple tumours from the Classic LFS (green), Chompret (blue) and HBC (orange) families. **A** The distribution of number of primary tumours in patients from these three family types. **B** The range of the time from first primary tumour to second primary tumour in patients from the three family phenotypes. Median time is six years to second primary.

Among the 112 diseased *TP53*-carriers, 35 patients (31%) had developed more than one primary tumour (range 2–5 tumours) (Fig. 4A), two patients belonged to “Other” (Table S3). The median time to the second primary tumour onset was 6 years in both the Classic LFS (range 2–32 years) and Chompret families (range 0-25 years), while it was 9 years among patients in the HBC families (range 0-25 years) (Fig. 4B).

### Genotype-phenotype correlation

**Fig. 5:**
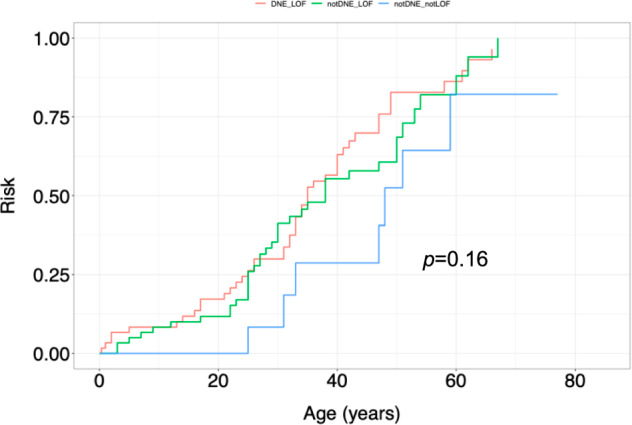
Cumulative cancer incidence stratified by variant classification. DNE dominant negative, LOF loss of function. *P* value: 0.16 (log-rank test).

Patients with variants classified as neither dominant-negative nor loss of function (notDNE_notLOF, Fig. 5) showed an overall lower cumulative lifetime cancer risk compared to other variants, although the difference was not statistically significant (*P* = 0.16, Fig. 5). We did not find significant differences (*p* = 0.17) between ages of tumour onset among affected carriers with missense variants (mean age 36) compared to carriers with other variants (mean age 31.5) (Table 3). The most common variant in our cohort, p.R181H, was found in 18 families, of whom four were classified as Chompret and the remaining as HBC. The youngest age of breast cancer onset was 29 years, in two families classified as Chompret. None of these families had any other tumour types reported. In the two remaining Chompret families, other tumours were in one family; squamous cell carcinoma, ovarian cancer and ovarian cancer in one family and in the other; brain tumour, bladder cancer, ovarian cancer, colorectal cancer and prostate cancer and non-Hodgkin lymphoma.

**Table 3:** Germline *TP53* variants: Spectrum and the comparison of age of onset based on variant types.

Variants types	No. of variants	No. of families	No. of families				No. of adult tumours No. of childhood		No. of patients	Mean/Median age (years)
			Classic LFS (13)	Chompret (37)	HBC (29)	Other (4)	Tumours			At first tumour (range, years)
**Missense**	22	58	6	24	24	3	65	12	74	36/36 (0.3–83)
Dominant-negative^a^	18	35	6	17	8	2	35	12	43	38/38 (0.3–83)
Not dominant-negative	2	4	0	2	1	1	9	0	7	47/51 (25–65)
Unclassified/no information	2	19	0	5	15	0	21	0	24	
**Other**										
Truncating	20	25	7	13	5	1	69	7	39	31.5/31 (2–71)
Nonsense	5	7	3	1	3	0	13	2	9	30/30 (3–52)
Frameshift deletion/duplication	8	10	1	8	0	1	24	3	13	34/31 (3–67)
Large deletion	2	2	0	0	2	0	6	0	4	57/62 (30–71)
Splicing	5	6	3	3	0	0	21	2	13	25/25 (12–40)

^a^Classified as DNE_LOF according to Giacomelli et al. [16].

The majority of childhood tumours 63% (12/19) were found in carriers of missense variants, all classified as dominant-negative (DNE_LOF) according to the IARC database (R20, February 2023), while 37% (7/19) of the tumours were identified in carriers with truncating variants (Table S4).

### De novo variants

Five patients were identified as carriers of a de novo variant, either after negative predictive testing of both parents or, for mosaic cases, if clonal haematopoiesis (CHIP) had been excluded by testing a second tissue and/or if a child inherited the variant. Five additional patients had a clinically assumed de novo variant: i.e. both parents were healthy, but unfortunately they were not available for testing or, for mosaic variants, a patient history indicative of hereditary *TP53*, but CHIP was not formally excluded in a second tissue (see column PM6, Table S5).

The genotype-phenotype correlation outcome of missense variants in this national cohort was also used to evaluate our previously published prediction model [12] for *TP53* missense variants and their association to a LFS phenotype rather than HBC (Table 1). Based on the available extended pedigree information from all families, the phenotypic presentation associated with each missense variant was interpreted: (1) as LFS if the family group Classic LFS/Chompret was more common than the number of reported HBC families for the same variant, (2) as HBC if the reported family group HBC was larger than the number of reported Classic LFS/Chompret families, and (3) as not informative if the number of reported Classic LFS/Chompret families and HBC families were equal, or if it was exclusively reported in the group of “Other”. Among the 22 identified missense variants two were considered as phenotypically not informative (families reported with both HBC and LFS, and one family belonging to the “Other” group, respectively) Table 1, 20 missense variants remained for prediction. Using our phenotypic in silico prediction model based on protein folding, using a threshold value set to of 0.65 (on a scale from 0 to 1), 15/20 (75%) variants (38 families) were predicted in agreement with the reported pedigree phenotype (i.e. correctly). If the threshold was set to 0.5 or 0.7, the number of correctly predicted families was lower, i.e. 65% and 70%, respectively (Table 1). Therefore, the threshold value of our prediction model was calibrated to 0.65 instead 0.5, as originally reported.

## Discussion

The aim of this study was to describe phenotypic characteristics of families with a clinically actionable (class 4 and 5) germline *TP53 (gTP53)* variant with regards to fulfilment of the genetic screening criteria for Classic Li-Fraumeni syndrome, Chompret or hereditary breast cancer, and to outline the genotype-phenotype correlation. The results were also used to evaluate our previously published phenotype prediction model for *TP53* missense variants and the likelihood for a carrier to develop an LFS-phenotype in relation to HBC.

Several attempts have been made to identify predictors of cancer risk for *TP53*-carriers [3, 9, 14], to explain the wide phenotypic variation in families with h*TP53*rc syndrome. It has partly been explained by different types of *TP53* variants; In a French cohort including 322 affected carriers, patients with missense variants, and in particular dominant-negative missense, showed an earlier age of tumour onset (23.8 years and 21.3 years, respectively) compared to those with loss of function variants (28.5 years) [9]. De Andrade et al. (2021) found that loss-of-function variants (with or without DNE) had an earlier age of onset and higher tumour risk [4]. Similarly, we found that carriers in our cohort with a DNE_LOF or notDNE_LOF variant had a tendency towards higher cumulative cancer incidence and earlier age of onset than those with other variants. We have previously made an attempt to further predict the phenotypic outcome of missense *TP53*-carriers by developing a prediction model based on their predicted impact on TP53 protein conformation [12]. By setting the threshold on the prediction model to 0.65 (on a scale 0–1) on this cohort, the correct phenotypic prediction of LFS in relation to HBC was 75% (Table 1). This tool could therefore be considered as useful in genetic counselling of families with h*TP53*rc. The psychological burden associated with genetic testing and participation in surveillance programmes may potentially be decreased if patients could be informed about lower risk of LFS in relation to HBC. However, the knowledge of penetrance prediction factors is still limited and individualised risk stratification of surveillance is therefore not suitable.

The most common variant in our cohort was the missense p.R181H, that was associated with mainly an HBC phenotype. Notably, in the GnomAD database (version 4.0 [15]) 24 healthy carriers (21/24 within Europe) with this variant are reported, and the IARC database report eight families (December 2023) with this variant (one in Norway, France, Germany, UK, China and Korea, and two in the USA). Our cohort with 18 families represents the largest published phenotype outcome of p.R181H and we therefore suggest that it may be a potential Swedish founder variant. Even though the p.R181H variant seems to be mostly associated with a breast cancer risk (identified in 14 HBC families and 4 Chompret families), it is not clear if we can restrict the surveillance to only breast cancer. Therefore, we are in the process of collecting additional clinical data to further outline the phenotypical characteristics of these patients.

One limitation to this type of phenotypic characterisation of pedigree cohort data is that families may diverge from one clinical category group to another as carriers develop new tumours. For example, a family categorised as HBC may be reclassified as Classic LFS or Chompret criteria family if a member develops a *TP53* associated tumour. Thus, long follow-up, and family size, is of importance, especially with regards to the HBC group in our study. Notably, among the eleven families that were identified between the years 2000-2011, six accounted for Classic LFS and five fulfilled the Chompret criteria, none met the HBC-criteria. There is a predominance of HBC families among the most recently analysed that could be partly due to the extended screening criteria (after 2012), but also due to shorter follow-up time. Therefore, conclusions concerning this group must be drawn with caution. Furthermore, for the purpose of this research study, we followed strict criteria for classification according to Chompret criteria which for instance only include breast cancer before 31 years. Therefore, the patient with bilateral DCIS at 26 years was classified as “Other”, although in a clinical real-life setting, we would of course offer her genetic testing for *TP53*. As only four patients were classified as “Other”, this does not impact on our overall results.

Regarding reclassification of variants of uncertain significance, even if a variant does not fulfil the ACMG criteria for pathogenicity, additional data from for example functional analyses and multiple families can in the future alter the classification. A final limitation using clinical testing data, is that older test results may also have reported CHIP variants with high variant allele frequency as germline variants. We did not have the possibility to formally rule out CHIP as a putative confounder in the patients with no other tested individuals in the family and who did not fulfil Classic LFS or Chompret criteria (such as patient 108 with ovarian cancer). However, we believe that these cases with CHIP will be few and should not affect the main conclusions from this study.

In conclusion, a characterisation of the Swedish cohort of known *TP53*-carriers identified 83 families with a clinically relevant variant. Of these families, 60% (50/83) of fulfilled the LFS or Chompret criteria for germline *TP53* screening and 35% (29/83) met the screening criteria for HBC. The missense *TP53* variant p.R181H was detected in 22% (18/83) of the presented Swedish families, and in 48% (14/29) of HBC, and was therefore identified as a potential Swedish founder predominantly associated with an HBC-phenotype. An evaluation of our previously published prediction model for carriers of missense g*TP53* variants correctly predicted the phenotypic outcome of LFS in relation to HBC in 75%. However, a substantial genotype-phenotype overlap remains, and it is therefore too early to individualise and stratify surveillance, thus all *TP53*-carriers should still be offered the same surveillance recommendations.

## Supplementary information

Supplementary Tables S1-S5.

Related Manuscript File

## Data Availability

The data generated for this paper can be found within the published paper and its supplementary file. Novel variants have been submitted to ClinVar (submission number SUB14867261).
